# Citizen science yields first records of *Hippocampus japapigu* and *Hippocampus denise* (Syngnathidae) from Taiwan: A hotspot for pygmy seahorse diversity

**DOI:** 10.3897/zookeys.883.39662

**Published:** 2019-10-28

**Authors:** Joseph Heard, Jeng-Ping Chen, Colin K.C. Wen

**Affiliations:** 1 Department of Life Science, Tunghai University, Taichung, Taiwan; 2 Taiwan Ocean Research Institute, National Applied Research Laboratories, Taiwan; 3 Center for Ecology and Environment, Tunghai University, Taiwan

**Keywords:** biodiversity monitoring, social media, web-based photographs

## Abstract

Relatively very little is known about pygmy seahorses, and even basic information regarding their distributions is largely inconsistent and often based on unofficial reports. However, monitoring marine diversity, particularly for small and cryptic species, such as pygmy seahorses, can be both costly and time consuming. In such cases, the use of citizen science can offer an effective tool for addressing knowledge gaps caused by a lack of biodiversity-related data. Scuba divers and underwater photographers were engaged through social media in order to investigate pygmy seahorse diversity in Taiwan. Using this approach five species of pygmy seahorses were identified, including two new records for Taiwan: *Hippocampus denise* and *Hippocampus japapigu*, the latter of which is the first record of the species from outside of Japan. These new records mark Taiwan as one of the world’s pygmy seahorse diversity hotspots, matching that of Japan and Indonesia, as well as demonstrating the value of citizen science for marine biodiversity monitoring, particularly for small cryptic species.

## Introduction

There are currently seven species of pygmy seahorse contained within the syngnathid genus *Hippocampus* Rafinesque, 1810 (Lourie et al. 2016). Diminutive sizes are a key feature among this unofficial grouping, ranging from 13.6 mm SL in *H. satomiae* Lourie & Kuiter, 2008 to 26.9 mm in *H. colemani* Kuiter, 2003 (Short et al. 2018). They can also be further differentiated from their congeners in possessing a single gill opening as opposed to a pair of openings, as well as trunk brooding rather than pouch brooding their young (Short et al. 2018).

The majority of pygmy seahorse species are known from a limited number of locations. For example, *H. waleananus* Gomon & Kuiter, 2008 is known only from Walea Island, Indonesia. As such, there is a severe paucity of information regarding various aspects of their ecology and biology. Basic occurrence data is also either lacking or inconsistent between online ichthyological database resources. Consequently, with the exceptions of *H. pontohi* Lourie & Kuiter, 2008 and *H. japapigu* Short et al., 2018, all other pygmy seahorse species are currently classified as “Data Deficient” on the IUCN Red List of Threatened Species. The latter, having only recently been described, has yet to be included.

Scientists are now increasingly and effectively utilising citizen science and other non-invasive methods to address knowledge gaps caused by constraints associated with the collection of biodiversity-related information (i.e., time and resources) (Miyazaki et al. 2014, 2015; Castilla et al. 2017; Campbell and Engelbrecht 2018; Pearson 2018). Social media (e.g., Facebook and Twitter), for example, has even been used to identify undescribed species (Skejo and Caballero 2016), as well as detecting illegal introductions of non-native species (Miyazaki et al. 2016). Such approaches are likely to prove particularly useful for small-sized, highly camouflaged cryptic taxa such as pygmy seahorses and sea slugs (Paz-Sedano et al. 2019). Recognising this, we engaged underwater photographers and dive guides through citizen science via social media in order to improve the current knowledge of pygmy seahorse diversity in Taiwan, where three species: *H. bargibanti* Whitely, 1970, *H. colemani*, and *H. pontohi* have so far been observed by scuba divers (Shao et al. 2008; Short et al. 2018).

## Materials and methods

We performed searches of “Posts” and “Photos” between 2017 and 2019 containing pygmy seahorses using the keyword “豆丁海馬” (pygmy seahorse in Chinese) from Facebook and Instagram, the most popular forms of social media used in Taiwan. For Facebook, three different user accounts were used in order to broaden potential search results due to Facebook’s algorithm. Manual searchers of a number of Taiwanese underwater photography and marine organism identification groups were also performed to generate additional sighting data. Individual users who frequently shared photographs of pygmy seahorses were also contacted directly to inquire about any additional sightings they may have made. The species, location, date, and depth (where available) were recorded for all photographs of pygmy seahorses taken within Taiwan.

## Results and discussion

**Figure 1.**
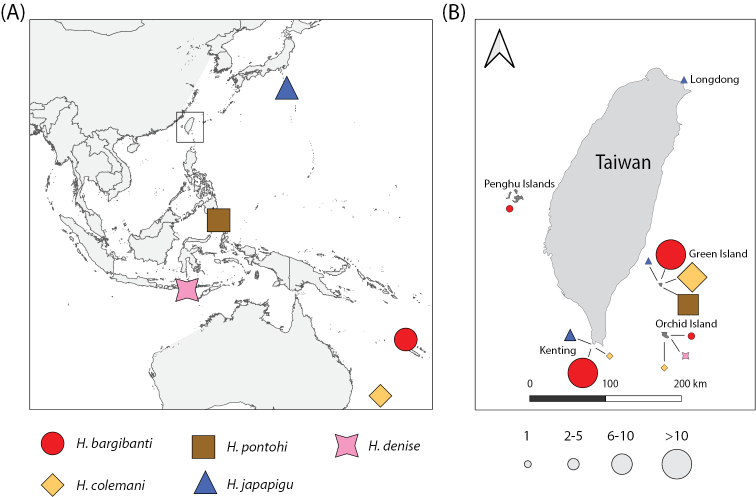
Map showing **A** the original collection locations of specimens for the five pygmy seahorse species recorded in Taiwan during this study, as well as **B** their distributions in Taiwan and surrounding islands (Penghu islands, Green Island, and Orchid Island). Symbols are scaled relatively according to the number of observations per species at each location obtained through social media.

**Figure 2.**
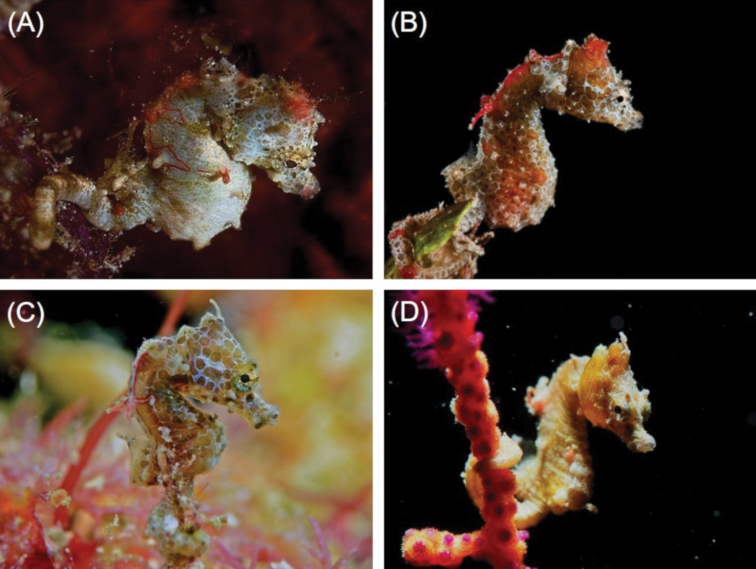
*Hippocampus japapigu* in situ **A** Green Island, Taiwan **B** Hejie, Kenting, Taiwan at 5 m depth **C** Hejie, Kenting, Taiwan **D** 82.5 k near Longdong, northern Taiwan (Photographs **A** Jolly Huang **B** Jay Chiu **C** Chao-Tsung Chen **D** Jung-Chao Yeh).

Our search results returned 259 social media items, 75 of which included in situ photographs of pygmy seahorses from five different locations in Taiwan (Fig. 1). From these we were able to identify 78 individuals belonging to five species (*H. bargibanti*, *H. colemani* and *H. pontohi*), including two new records for Taiwan (*H. denise* and *H. japapigu*). Firstly, we report the first record of *H. japapigu* from Taiwan based on four separate in situ observations, which also represent the first records of the species from outside of Japan. The earliest observation of what appears to be *H. japapigu* was photographed at Green Island in 2010 (Fig. 2A). This was mistakenly lauded on social media as Taiwan’s first record of *H. pontohi*, the closest congener of *H. japapigu*, which had yet to be described at the time. A group of seven *H. japapigu* were later observed in association with *Halimeda* algae at 5 m during a night dive in Hejie, Kenting in southern Taiwan in 2017 (Fig. 2B). These were again mistaken by the photographer as *H. pontohi*; however, in hindsight we can now confirm these individuals were *H. japapigu* based on their distinctive reticulated white patterning, single pair of bilaterally paired wing-like protrusions, raised dorsal ridge, as well as a pronounced eighth lateral trunk ridge spine (Short et al. 2018). Further sightings have since been reported, with a single individual observed again in Hejie in 2017 (Fig. 2C), as well as a single individual in Longdong in northern Taiwan in 2019 (Fig. 2D).

**Figure 3.**
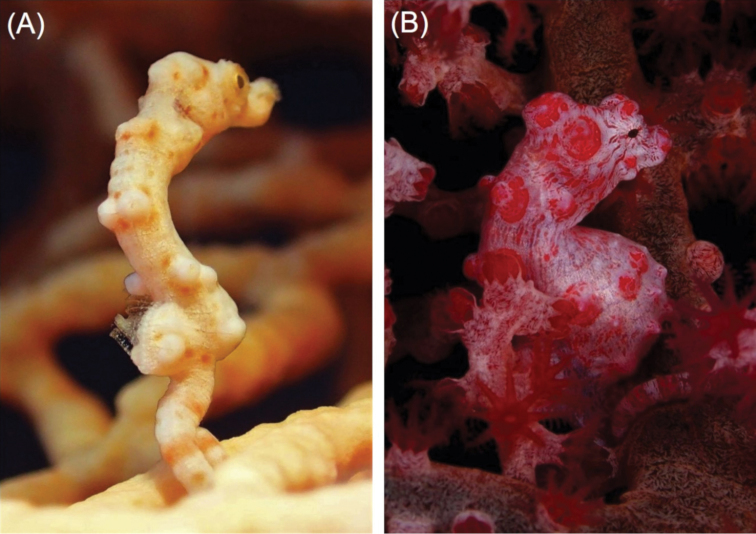
Comparison of **A***Hippocampus denise* in situ, Orchid Island, Taiwan at 28 m depth, with its most similar congener **B***Hippocampus bargibanti* in situ, Green Island, Taiwan. Note the differences in body colouration (orange in *H. denise* vs. purple in *H. bargibanti*), the number and size of tubercles (fewer and less pronounced in *H. denise*), snout length (bulbous tip in *H. bargibanti* vs. non-bulbous in *H. denise*) and overall shape (slender and elongate in *H. denise* vs. rotund in *H. bargibanti*) (Photographs **A** Yung-Kuang Ting **B** Ryan Ku).

We also report the first finding of *H. denise* from Taiwan, which is one of the smallest and most widely distributed of the pygmy seahorses, occurring throughout much of the Indo-West Pacific (Lourie and Randall 2003; Foster et al. 2011). A single female was observed inhabiting the branches of an *Annella* gorgonian coral at a depth of 28 m at Orchid Island (Lanyu), off the southeast coast of Taiwan (Fig. 3A). This species can be easily distinguished from its nearest congener, *H. bargibanti* (Fig. 3B), based on its fewer and less developed tubercles, orange body colouration, non-bulbous snout and slender and elongated body shape, the latter of which was the most frequently recorded and widely distributed species in this study. This is unsurprising given their conspicuousness and larger size relative to the majority of other pygmy seahorses. Indeed, *H. bargibanti* was the first species to be recorded in Taiwan, having initially been observed in Kenting in 2004, and to date remains the only species to have been formally documented (Shao et al. 2008).

Lastly, we also confirm the presence of *H. colemani* and *H. pontohi* (*H. severnsi* is a junior synonym of the latter) from Taiwan based on numerous observations. With the exceptions of single sightings of *H. colemani* from both Orchid Island and Kenting, *H. colemani* was predominantly observed at Green Island, where it was the most commonly sighted species. Conversely, *H. pontohi* was only recorded from Green Island. The two species can be visually differentiated based on the low and rounded coronet of *H. colemani* (Fig. 3A–C), which is more distinct and angular in *H. pontohi* (Fig. 3D–F) (Short et al. 2018).

As five of the known seven species of pygmy seahorses have been observed in Taiwan, the country now ranks as one of the world’s pygmy seahorse diversity hotspots. Of particular note, four species were found at Green Island alone, a small island measuring only 15.09 km^2^. However, no voucher specimens of any pygmy seahorse species have so far been collected from Taiwan. This is unfortunate given the importance of scientific collections for studies of evolution, ecology, and conservation (Rocha et al. 2014). We therefore recommend the collection of specimens from Taiwan to facilitate further research into these poorly understood taxa.

**Figure 4.**
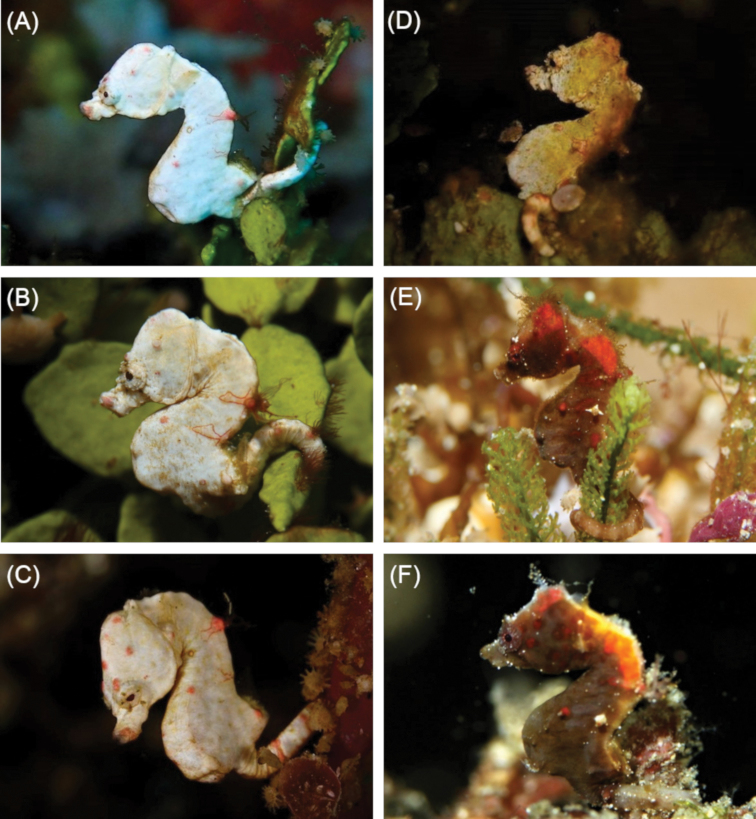
Comparison of **A–C***Hippocampus colemani* in situ, Green Island, Taiwan with **D–F***Hippocampus pontohi* in situ, Green Island, Taiwan. Note the differences in the shape and angle of the coronet (low and rounded in *H. colemani* vs. distinct and angular in *H. pontohi*), as well as differences in body colouration (*H. colemani* is known only to occur in shades of off-white, whereas *H. pontohi* is highly variable) (Photographs **A** Joe Chiu **B** Ryan Ku **C, E, F** Ming-Hung Yu **D** Ryan Ku).
